# 5-Isobutyl-4-phenyl­sulfonyl-1*H*-pyrazol-3(2*H*)-one

**DOI:** 10.1107/S1600536810044181

**Published:** 2010-11-06

**Authors:** Wan-Sin Loh, Hoong-Kun Fun, R. Venkat Ragavan, V. Vijayakumar, M. Venkatesh

**Affiliations:** a X-ray Crystallography Unit, School of Physics, Universiti Sains Malaysia, 11800 USM, Penang, Malaysia; b Organic Chemistry Division, School of Advanced Sciences, VIT University, Vellore 632 014, India

## Abstract

The title compound, C_13_H_16_N_2_O_3_S, consists of two crystallographically independent mol­ecules with similar geometries and exists in a keto form, the C=O bond lengths being 1.267 (2) and 1.254 (2) Å. In both mol­ecules, the pyrazole rings are approximately planar, with maximum deviations of 0.017 (2) and 0.010 (2) Å, and the dihedral angles between the pyrazole and phenyl rings are 83.63 (11) and 70.07 (12)°. In one mol­ecule, an intra­molecular C—H⋯O hydrogen bond with an *S*(6) ring motif is observed. In the crystal, inter­molecular N—H⋯O and C—H⋯O hydrogen bonds link the mol­ecules into two-dimensional networks parallel to the *ab* plane.

## Related literature

For background to pyrazole derivatives and their microbial activities, see: Ragavan *et al.* (2009 ▶, 2010 ▶). For bond-length data, see: Allen *et al.* (1987 ▶). For related structures, see: Loh, Fun, Ragavan, Vijayakumar & Sarveswari (2010 ▶); Loh, Fun, Ragavan, Vijayakumar & Venkatesh (2010 ▶); Shahani *et al.* (2010 ▶). For hydrogen-bond motifs, see: Bernstein *et al.* (1995 ▶). For the stability of the temperature controller used for the data collection, see: Cosier & Glazer (1986 ▶).
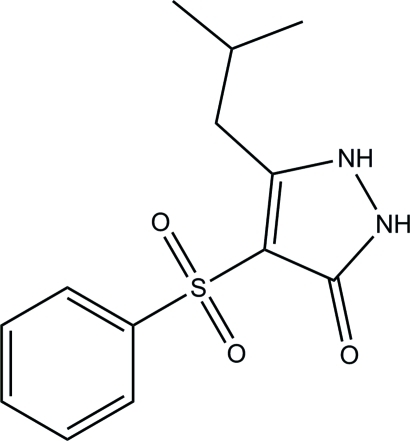


## Experimental

### 

#### Crystal data

C_13_H_16_N_2_O_3_S
*M* *_r_* = 280.34
Triclinic, 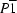

*a* = 11.3423 (8) Å
*b* = 11.9987 (9) Å
*c* = 12.4657 (9) Å
α = 98.579 (3)°
β = 113.038 (3)°
γ = 112.882 (3)°
*V* = 1344.42 (17) Å^3^
*Z* = 4
Mo *K*α radiation
μ = 0.25 mm^−1^
*T* = 100 K
0.56 × 0.20 × 0.18 mm

#### Data collection

Bruker SMART APEXII CCD area-detector diffractometer
Absorption correction: multi-scan (*SADABS*; Bruker, 2009 ▶) *T*_min_ = 0.875, *T*_max_ = 0.958
18458 measured reflections
5202 independent reflections
4816 reflections with *I* > 2σ(*I*)
*R*_int_ = 0.034

#### Refinement

*R*[*F* ^2^ > 2σ(*F* ^2^)] = 0.036
*wR*(*F* ^2^) = 0.109
*S* = 1.04
5202 reflections
363 parameters
H atoms treated by a mixture of independent and constrained refinement
Δρ_max_ = 0.67 e Å^−3^
Δρ_min_ = −0.38 e Å^−3^

### 

Data collection: *APEX2* (Bruker, 2009 ▶); cell refinement: *SAINT* (Bruker, 2009 ▶); data reduction: *SAINT*; program(s) used to solve structure: *SHELXTL* (Sheldrick, 2008 ▶); program(s) used to refine structure: *SHELXTL*; molecular graphics: *SHELXTL*; software used to prepare material for publication: *SHELXTL* and *PLATON* (Spek, 2009 ▶).

## Supplementary Material

Crystal structure: contains datablocks global, I. DOI: 10.1107/S1600536810044181/is2619sup1.cif

Structure factors: contains datablocks I. DOI: 10.1107/S1600536810044181/is2619Isup2.hkl

Additional supplementary materials:  crystallographic information; 3D view; checkCIF report

## Figures and Tables

**Table 1:** Hydrogen-bond geometry (Å, °)

*D*—H⋯*A*	*D*—H	H⋯*A*	*D*⋯*A*	*D*—H⋯*A*
N1*A*—H1*NA*⋯O3*A*^i^	0.79 (3)	2.05 (3)	2.816 (2)	164 (3)
N2*A*—H2*NA*⋯O3*B*^ii^	0.85 (4)	1.85 (4)	2.640 (3)	155 (2)
N1*B*—H1*NB*⋯O1*A*^iii^	0.86 (3)	2.10 (4)	2.733 (3)	130 (3)
N2*B*—H2*NB*⋯O3*A*^iii^	0.88 (4)	1.83 (4)	2.700 (3)	170 (2)
C5*A*—H5*AA*⋯O1*B*^iv^	0.93	2.47	3.256 (3)	143
C5*B*—H5*BA*⋯O2*A*	0.93	2.48	3.307 (3)	149
C10*A*—H10*B*⋯O3*B*^ii^	0.97	2.57	3.324 (3)	135
C10*B*—H10*D*⋯O1*B*	0.97	2.41	3.152 (3)	133

Symmetry codes: (i) 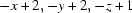; (ii) 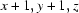; (iii) 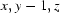; (iv) 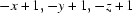.

**Fig. 1.**
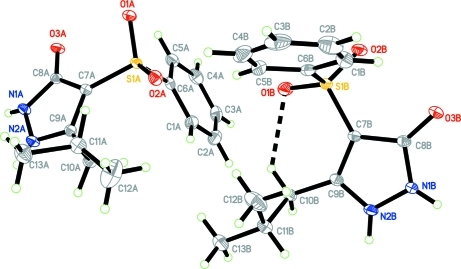
The molecular structure of the title compound, showing 50% probability displacement ellipsoids and the atom-numbering scheme. The dashed line indicates the intramolecular hydrogen bond.

**Fig. 2.**
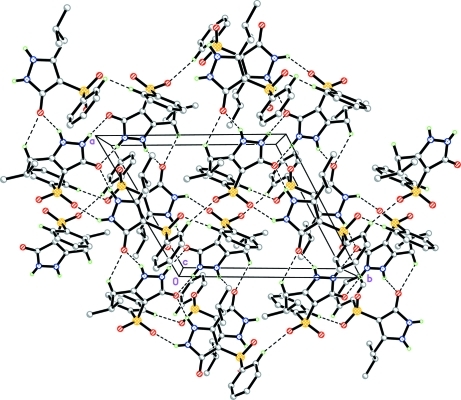
The crystal packing of the title compound, showing two-dimensional networks parallel to the ab plane. H atoms not involved in the intermolecular interactions (dashed lines) have been omitted for clarity.

## supplementary crystallographic information

### Comment

Antibacterial and antifungal activities of the azoles are most widely studied and some of them are in clinical practice as anti-microbial agents. However, the azole-resistant strains had led to the development of new anti-microbial compounds. In particular, pyrazole derivatives are extensively studied and used as anti-microbial agents. Pyrazole is an important class of heterocyclic compounds and many pyrazole derivatives are reported to have the broad spectrum of biological properties such as anti-inflammatory, antifungal, herbicidal, anti-tumour, cytotoxic, molecular modelling and antiviral activities. Pyrazole derivatives also act as anti-angiogenic agents, A3 adenosine receptor antagonists, neuropeptide YY5 receptor antagonists as well as kinase inhibitor for treatment of type 2 diabetes, hyperlipidemia, obesity and thrombopiotinmimetics. Recently urea derivatives of pyrazoles have been reported as potent inhibitors of p38 kinase. Since the high electronegativity of halogens (particularly chlorine and fluorine) in the aromatic part of the drug molecules play an important role in enhancing their biological activity, we are interested to have 4-fluoro or 4-chloro substitution in the aryls of 1,5-diaryl pyrazoles. As part of our on-going research aiming the synthesis of new anti-microbial compounds, we have reported the synthesis of novel pyrazole derivatives and their microbial activities (Ragavan *et al.*, 2009, 2010).

The title compound consists of two crystallographically independent molecules, with similar geometries, namely molecules *A* and *B* and exist in keto-form. This indicates that the compound undergoes an enol-to-keto tautomerism during the crystallization process with the bond lengths of C═ O being 1.267 (2) and 1.254 (2) Å in molecule *A* and *B*, respectively. In molecule *A*, the pyrazole ring (C7A/C8A/N1A/N2A/C9A) is approximately planar with a maximum deviation of 0.017 (2) Å at atom C7A and almost perpendicular with the phenyl ring (C1A–C6A) with a dihedral angle of 83.63 (11)°. In molecule *B*, the pyrazole ring (C7B/C8B/N1B/N2B/C9B) with a maximum deviation being 0.010 (2) Å at C8B forms a dihedral angle of 70.07 (12)° with the phenyl ring (C1B–C6B) and further stabilized by an *S*(6) ring motif (Bernstein *et al.*, 1995) *via* the intramolecular C10B—H10D···O1B hydrogen bond. Bond lengths (Allen *et al.*, 1987) and angles are within the normal ranges and are comparable to the related structures (Loh, Fun, Ragavan, Vijayakumar & Sarveswari, 2010; Loh, Fun, Ragavan, Vijayakumar & Venkatesh, 2010; Shahani *et al.*, 2010).

In the crystal packing, intermolecular N1A—H1NA···O3A, N2A—H2NA···O3B, N1B—H1NB···O1A, N2B—H2NB···O3A, C5A—H5AA···O1B, C5B—H5BA···O2A and C10A—H10B···O3B hydrogen bonds (Table 1) link the molecules into two-dimensional networks parallel to *ab* plane (Fig. 2).

### Experimental

3-Isobutyl-4-(phenylthiol)-1*H*-pyrazol-5-ol was synthesized using the method available in the literature (Ragavan *et al.*, 2010). It was then dissolved in 1:1 mixture of THF/Water. Oxone was then added and the solution was stirred at room temperature for 3 h. The reaction mixture was diluted with water (20 ml) and then extracted with ethylacetate (2 *x* 50 ml). The combined extract was washed with water (20 ml) and brine solution. The titled compound was recrystallized using the ethanol-chloroform 1:1 mixture. Yield: 50%. *M. p.* = 487–489 K.

### Refinement

N-bound H atoms were located in a difference Fourier map and was refined freely [N—H = 0.79 (3) to 0.88 (3) Å]. The remaining H atoms were positioned geometrically with the bond length of C—H being 0.93 to 0.98 Å and were refined using a riding model, with *U*_iso_(H) = 1.2 or 1.5 *U*_eq_(C). A rotating group model was applied to the methyl groups.

### Crystal data

**Table:**

C_13_H_16_N_2_O_3_S	*Z* = 4
*M**_r_* = 280.34	*F*(000) = 592
Triclinic, *P*1	*D*_x_ = 1.385 Mg m^−^^3^
Hall symbol: -P 1	Mo *K*α radiation, λ = 0.71073 Å
*a* = 11.3423 (8) Å	Cell parameters from 9939 reflections
*b* = 11.9987 (9) Å	θ = 2.6–35.0°
*c* = 12.4657 (9) Å	µ = 0.25 mm^−^^1^
α = 98.579 (3)°	*T* = 100 K
β = 113.038 (3)°	Block, colourless
γ = 112.882 (3)°	0.56 × 0.20 × 0.18 mm
*V* = 1344.42 (17) Å^3^	

### Data collection

**Table:**

Bruker SMART APEXII CCD area-detector diffractometer	5202 independent reflections
Radiation source: fine-focus sealed tube	4816 reflections with *I* > 2σ(*I*)
graphite	*R*_int_ = 0.034
φ and ω scans	θ_max_ = 26.0°, θ_min_ = 1.9°
Absorption correction: multi-scan (*SADABS*; Bruker, 2009)	*h* = −13→13
*T*_min_ = 0.875, *T*_max_ = 0.958	*k* = −14→14
18458 measured reflections	*l* = −15→15

### Refinement

**Table:**

Refinement on *F*^2^	Primary atom site location: structure-invariant direct methods
Least-squares matrix: full	Secondary atom site location: difference Fourier map
*R*[*F*^2^ > 2σ(*F*^2^)] = 0.036	Hydrogen site location: inferred from neighbouring sites
*wR*(*F*^2^) = 0.109	H atoms treated by a mixture of independent and constrained refinement
*S* = 1.04	*w* = 1/[σ^2^(*F*_o_^2^) + (0.0553*P*)^2^ + 1.2051*P*] where *P* = (*F*_o_^2^ + 2*F*_c_^2^)/3
5202 reflections	(Δ/σ)_max_ = 0.001
363 parameters	Δρ_max_ = 0.67 e Å^−^^3^
0 restraints	Δρ_min_ = −0.38 e Å^−^^3^

### Special details

**Table:**

Experimental. The crystal was placed in the cold stream of an Oxford Cryosystems Cobra open-flow nitrogen cryostat (Cosier & Glazer, 1986) operating at 100.0 (1) K.
Geometry. All e.s.d.'s (except the e.s.d. in the dihedral angle between two l.s. planes) are estimated using the full covariance matrix. The cell e.s.d.'s are taken into account individually in the estimation of e.s.d.'s in distances, angles and torsion angles; correlations between e.s.d.'s in cell parameters are only used when they are defined by crystal symmetry. An approximate (isotropic) treatment of cell e.s.d.'s is used for estimating e.s.d.'s involving l.s. planes.
Refinement. Refinement of *F*^2^ against ALL reflections. The weighted *R*-factor *wR* and goodness of fit *S* are based on *F*^2^, conventional *R*-factors *R* are based on *F*, with *F* set to zero for negative *F*^2^. The threshold expression of *F*^2^ > σ(*F*^2^) is used only for calculating *R*-factors(gt) *etc*. and is not relevant to the choice of reflections for refinement. *R*-factors based on *F*^2^ are statistically about twice as large as those based on *F*, and *R*- factors based on ALL data will be even larger.

### Fractional atomic coordinates and isotropic or equivalent isotropic displacement parameters (Å^2^)

**Table:**

	*x*	*y*	*z*	*U*_iso_*/*U*_eq_	
S1A	0.61779 (4)	0.56939 (4)	0.30323 (4)	0.01287 (12)	
O1A	0.52206 (14)	0.62292 (12)	0.29583 (12)	0.0177 (3)	
O2A	0.56191 (14)	0.44944 (12)	0.20809 (11)	0.0176 (3)	
O3A	0.81177 (13)	0.88563 (12)	0.44040 (11)	0.0155 (3)	
N1A	0.96797 (17)	0.87383 (15)	0.36853 (14)	0.0144 (3)	
N2A	0.97863 (17)	0.77840 (15)	0.30426 (13)	0.0146 (3)	
C1A	0.7407 (2)	0.46688 (18)	0.46562 (17)	0.0190 (4)	
H1AA	0.7415	0.4199	0.3996	0.023*	
C2A	0.7970 (2)	0.45448 (19)	0.58116 (18)	0.0223 (4)	
H2AA	0.8369	0.3998	0.5934	0.027*	
C3A	0.7936 (2)	0.52380 (19)	0.67853 (17)	0.0221 (4)	
H3AA	0.8317	0.5157	0.7561	0.027*	
C4A	0.7335 (2)	0.60524 (19)	0.66063 (17)	0.0227 (4)	
H4AA	0.7299	0.6499	0.7259	0.027*	
C5A	0.6789 (2)	0.62047 (18)	0.54597 (17)	0.0192 (4)	
H5AA	0.6405	0.6762	0.5341	0.023*	
C6A	0.68325 (19)	0.55035 (16)	0.44963 (16)	0.0144 (3)	
C7A	0.77240 (19)	0.68499 (17)	0.31001 (15)	0.0137 (3)	
C8A	0.84468 (18)	0.82068 (16)	0.37900 (15)	0.0128 (3)	
C9A	0.86263 (19)	0.66367 (17)	0.26817 (15)	0.0132 (3)	
C10A	0.84569 (19)	0.54146 (17)	0.19624 (15)	0.0155 (3)	
H10A	0.8084	0.4743	0.2277	0.019*	
H10B	0.9418	0.5559	0.2107	0.019*	
C11A	0.7416 (2)	0.49321 (18)	0.05437 (16)	0.0190 (4)	
H11A	0.6510	0.4944	0.0407	0.023*	
C12A	0.7026 (3)	0.3539 (2)	−0.0044 (2)	0.0394 (6)	
H12A	0.6353	0.3223	−0.0922	0.059*	
H12B	0.6576	0.3006	0.0337	0.059*	
H12C	0.7905	0.3511	0.0084	0.059*	
C13A	0.8111 (2)	0.5804 (2)	−0.00512 (18)	0.0249 (4)	
H13A	0.7427	0.5505	−0.0924	0.037*	
H13B	0.8986	0.5784	0.0054	0.037*	
H13C	0.8358	0.6675	0.0341	0.037*	
S1B	0.32612 (5)	0.06328 (4)	0.30231 (4)	0.01364 (12)	
O1B	0.44172 (14)	0.19562 (12)	0.37062 (12)	0.0203 (3)	
O2B	0.20554 (14)	0.01636 (13)	0.32778 (12)	0.0184 (3)	
O3B	0.18314 (13)	−0.24470 (12)	0.26728 (12)	0.0177 (3)	
N1B	0.41780 (16)	−0.21360 (15)	0.33582 (14)	0.0156 (3)	
N2B	0.55364 (17)	−0.11548 (15)	0.36901 (14)	0.0158 (3)	
C1B	0.1298 (2)	−0.07887 (19)	0.05619 (17)	0.0221 (4)	
H1BA	0.0925	−0.1462	0.0827	0.026*	
C2B	0.0666 (2)	−0.0952 (2)	−0.06954 (19)	0.0300 (5)	
H2BA	−0.0139	−0.1743	−0.1280	0.036*	
C3B	0.1230 (3)	0.0058 (2)	−0.10850 (19)	0.0320 (5)	
H3BA	0.0792	−0.0057	−0.1928	0.038*	
C4B	0.2432 (3)	0.1227 (2)	−0.0231 (2)	0.0304 (5)	
H4BA	0.2810	0.1893	−0.0502	0.036*	
C5B	0.3091 (2)	0.14218 (19)	0.10415 (19)	0.0224 (4)	
H5BA	0.3904	0.2211	0.1623	0.027*	
C6B	0.2500 (2)	0.04043 (18)	0.14147 (16)	0.0163 (4)	
C7B	0.40072 (19)	−0.03787 (17)	0.32486 (15)	0.0135 (3)	
C8B	0.31688 (19)	−0.17245 (17)	0.30481 (15)	0.0141 (3)	
C9B	0.54630 (19)	−0.00867 (17)	0.36371 (15)	0.0139 (3)	
C10B	0.68105 (19)	0.11342 (17)	0.39661 (16)	0.0166 (4)	
H10C	0.7581	0.1324	0.4788	0.020*	
H10D	0.6592	0.1840	0.4009	0.020*	
C11B	0.7392 (2)	0.10832 (19)	0.30404 (17)	0.0207 (4)	
H11B	0.7586	0.0355	0.2987	0.025*	
C12B	0.6260 (2)	0.0862 (3)	0.1743 (2)	0.0346 (5)	
H12D	0.6646	0.0825	0.1185	0.052*	
H12E	0.6043	0.1560	0.1777	0.052*	
H12F	0.5376	0.0060	0.1449	0.052*	
C13B	0.8834 (2)	0.2320 (2)	0.3531 (2)	0.0273 (4)	
H13D	0.9217	0.2272	0.2973	0.041*	
H13E	0.9528	0.2424	0.4342	0.041*	
H13F	0.8666	0.3047	0.3588	0.041*	
H1NA	1.035 (3)	0.944 (3)	0.412 (2)	0.028 (6)*	
H2NA	1.054 (3)	0.795 (2)	0.296 (2)	0.032 (7)*	
H1NB	0.407 (3)	−0.289 (3)	0.332 (2)	0.026 (6)*	
H2NB	0.632 (3)	−0.125 (2)	0.390 (2)	0.026 (6)*	

### Atomic displacement parameters (Å^2^)

**Table:**

	*U*^11^	*U*^22^	*U*^33^	*U*^12^	*U*^13^	*U*^23^
S1A	0.0104 (2)	0.0121 (2)	0.0158 (2)	0.00489 (17)	0.00692 (16)	0.00437 (16)
O1A	0.0132 (6)	0.0178 (6)	0.0253 (6)	0.0090 (5)	0.0106 (5)	0.0082 (5)
O2A	0.0161 (6)	0.0136 (6)	0.0181 (6)	0.0045 (5)	0.0079 (5)	0.0026 (5)
O3A	0.0119 (6)	0.0154 (6)	0.0190 (6)	0.0073 (5)	0.0082 (5)	0.0027 (5)
N1A	0.0108 (7)	0.0123 (7)	0.0174 (7)	0.0041 (6)	0.0074 (6)	0.0020 (6)
N2A	0.0122 (7)	0.0164 (7)	0.0168 (7)	0.0072 (6)	0.0088 (6)	0.0043 (6)
C1A	0.0214 (9)	0.0169 (9)	0.0207 (9)	0.0096 (8)	0.0121 (8)	0.0060 (7)
C2A	0.0231 (10)	0.0194 (9)	0.0233 (9)	0.0108 (8)	0.0095 (8)	0.0090 (7)
C3A	0.0208 (10)	0.0221 (9)	0.0176 (8)	0.0054 (8)	0.0087 (7)	0.0091 (7)
C4A	0.0219 (10)	0.0241 (10)	0.0194 (9)	0.0075 (8)	0.0131 (8)	0.0039 (7)
C5A	0.0176 (9)	0.0176 (9)	0.0222 (9)	0.0073 (7)	0.0118 (7)	0.0047 (7)
C6A	0.0124 (8)	0.0133 (8)	0.0167 (8)	0.0040 (7)	0.0085 (7)	0.0060 (6)
C7A	0.0124 (8)	0.0143 (8)	0.0156 (8)	0.0069 (7)	0.0076 (7)	0.0056 (6)
C8A	0.0103 (8)	0.0158 (8)	0.0122 (7)	0.0069 (7)	0.0047 (6)	0.0058 (6)
C9A	0.0129 (8)	0.0157 (8)	0.0123 (7)	0.0077 (7)	0.0062 (6)	0.0063 (6)
C10A	0.0152 (8)	0.0156 (8)	0.0166 (8)	0.0080 (7)	0.0085 (7)	0.0046 (7)
C11A	0.0199 (9)	0.0199 (9)	0.0149 (8)	0.0090 (8)	0.0081 (7)	0.0038 (7)
C12A	0.0616 (17)	0.0238 (11)	0.0218 (10)	0.0181 (11)	0.0157 (11)	0.0026 (9)
C13A	0.0250 (10)	0.0301 (11)	0.0193 (9)	0.0120 (9)	0.0117 (8)	0.0098 (8)
S1B	0.0122 (2)	0.0146 (2)	0.0157 (2)	0.00772 (18)	0.00735 (17)	0.00464 (16)
O1B	0.0167 (7)	0.0157 (6)	0.0240 (6)	0.0081 (6)	0.0075 (5)	0.0024 (5)
O2B	0.0166 (6)	0.0225 (7)	0.0225 (6)	0.0122 (6)	0.0126 (5)	0.0085 (5)
O3B	0.0127 (6)	0.0190 (6)	0.0234 (6)	0.0082 (5)	0.0103 (5)	0.0073 (5)
N1B	0.0123 (7)	0.0151 (8)	0.0222 (7)	0.0075 (6)	0.0098 (6)	0.0079 (6)
N2B	0.0108 (7)	0.0185 (8)	0.0188 (7)	0.0082 (6)	0.0072 (6)	0.0062 (6)
C1B	0.0190 (9)	0.0246 (10)	0.0216 (9)	0.0102 (8)	0.0099 (8)	0.0078 (8)
C2B	0.0224 (10)	0.0404 (12)	0.0197 (9)	0.0156 (10)	0.0064 (8)	0.0032 (9)
C3B	0.0368 (12)	0.0576 (15)	0.0221 (10)	0.0364 (12)	0.0170 (9)	0.0209 (10)
C4B	0.0405 (13)	0.0420 (13)	0.0377 (11)	0.0315 (11)	0.0284 (10)	0.0290 (10)
C5B	0.0237 (10)	0.0226 (10)	0.0306 (10)	0.0150 (8)	0.0167 (8)	0.0140 (8)
C6B	0.0154 (9)	0.0207 (9)	0.0179 (8)	0.0123 (8)	0.0088 (7)	0.0083 (7)
C7B	0.0127 (8)	0.0155 (8)	0.0144 (8)	0.0081 (7)	0.0075 (6)	0.0055 (6)
C8B	0.0147 (9)	0.0182 (9)	0.0136 (8)	0.0097 (7)	0.0089 (7)	0.0061 (7)
C9B	0.0137 (8)	0.0173 (8)	0.0121 (7)	0.0078 (7)	0.0075 (7)	0.0050 (6)
C10B	0.0132 (8)	0.0172 (9)	0.0186 (8)	0.0069 (7)	0.0081 (7)	0.0055 (7)
C11B	0.0183 (9)	0.0235 (10)	0.0248 (9)	0.0110 (8)	0.0133 (8)	0.0098 (8)
C12B	0.0247 (11)	0.0568 (15)	0.0258 (10)	0.0180 (11)	0.0156 (9)	0.0195 (10)
C13B	0.0229 (10)	0.0261 (10)	0.0363 (11)	0.0103 (9)	0.0191 (9)	0.0120 (9)

### Geometric parameters (Å, °)

**Table:**

S1A—O2A	1.4397 (13)	S1B—O1B	1.4385 (13)
S1A—O1A	1.4428 (13)	S1B—O2B	1.4431 (13)
S1A—C7A	1.7215 (18)	S1B—C7B	1.7269 (17)
S1A—C6A	1.7691 (17)	S1B—C6B	1.7713 (18)
O3A—C8A	1.267 (2)	O3B—C8B	1.254 (2)
N1A—C8A	1.361 (2)	N1B—C8B	1.362 (2)
N1A—N2A	1.370 (2)	N1B—N2B	1.370 (2)
N1A—H1NA	0.79 (3)	N1B—H1NB	0.86 (3)
N2A—C9A	1.331 (2)	N2B—C9B	1.326 (2)
N2A—H2NA	0.84 (3)	N2B—H2NB	0.88 (3)
C1A—C2A	1.386 (3)	C1B—C2B	1.389 (3)
C1A—C6A	1.390 (3)	C1B—C6B	1.390 (3)
C1A—H1AA	0.9300	C1B—H1BA	0.9300
C2A—C3A	1.388 (3)	C2B—C3B	1.389 (3)
C2A—H2AA	0.9300	C2B—H2BA	0.9300
C3A—C4A	1.390 (3)	C3B—C4B	1.375 (3)
C3A—H3AA	0.9300	C3B—H3BA	0.9300
C4A—C5A	1.390 (3)	C4B—C5B	1.398 (3)
C4A—H4AA	0.9300	C4B—H4BA	0.9300
C5A—C6A	1.389 (2)	C5B—C6B	1.391 (3)
C5A—H5AA	0.9300	C5B—H5BA	0.9300
C7A—C9A	1.402 (2)	C7B—C9B	1.399 (2)
C7A—C8A	1.433 (2)	C7B—C8B	1.440 (2)
C9A—C10A	1.498 (2)	C9B—C10B	1.496 (2)
C10A—C11A	1.544 (2)	C10B—C11B	1.541 (2)
C10A—H10A	0.9700	C10B—H10C	0.9700
C10A—H10B	0.9700	C10B—H10D	0.9700
C11A—C13A	1.521 (3)	C11B—C13B	1.520 (3)
C11A—C12A	1.528 (3)	C11B—C12B	1.522 (3)
C11A—H11A	0.9800	C11B—H11B	0.9800
C12A—H12A	0.9600	C12B—H12D	0.9600
C12A—H12B	0.9600	C12B—H12E	0.9600
C12A—H12C	0.9600	C12B—H12F	0.9600
C13A—H13A	0.9600	C13B—H13D	0.9600
C13A—H13B	0.9600	C13B—H13E	0.9600
C13A—H13C	0.9600	C13B—H13F	0.9600
			
O2A—S1A—O1A	119.07 (8)	O1B—S1B—O2B	118.68 (8)
O2A—S1A—C7A	108.50 (8)	O1B—S1B—C7B	109.29 (8)
O1A—S1A—C7A	107.89 (8)	O2B—S1B—C7B	106.75 (8)
O2A—S1A—C6A	108.27 (8)	O1B—S1B—C6B	107.72 (8)
O1A—S1A—C6A	107.77 (8)	O2B—S1B—C6B	107.22 (8)
C7A—S1A—C6A	104.39 (8)	C7B—S1B—C6B	106.56 (8)
C8A—N1A—N2A	109.89 (14)	C8B—N1B—N2B	110.67 (15)
C8A—N1A—H1NA	123.5 (19)	C8B—N1B—H1NB	130.6 (16)
N2A—N1A—H1NA	123.7 (19)	N2B—N1B—H1NB	118.5 (16)
C9A—N2A—N1A	110.01 (15)	C9B—N2B—N1B	109.61 (15)
C9A—N2A—H2NA	128.6 (17)	C9B—N2B—H2NB	126.7 (16)
N1A—N2A—H2NA	121.2 (17)	N1B—N2B—H2NB	123.7 (16)
C2A—C1A—C6A	119.19 (16)	C2B—C1B—C6B	118.48 (19)
C2A—C1A—H1AA	120.4	C2B—C1B—H1BA	120.8
C6A—C1A—H1AA	120.4	C6B—C1B—H1BA	120.8
C1A—C2A—C3A	119.87 (18)	C3B—C2B—C1B	120.4 (2)
C1A—C2A—H2AA	120.1	C3B—C2B—H2BA	119.8
C3A—C2A—H2AA	120.1	C1B—C2B—H2BA	119.8
C2A—C3A—C4A	120.31 (17)	C4B—C3B—C2B	120.35 (19)
C2A—C3A—H3AA	119.8	C4B—C3B—H3BA	119.8
C4A—C3A—H3AA	119.8	C2B—C3B—H3BA	119.8
C3A—C4A—C5A	120.56 (17)	C3B—C4B—C5B	120.6 (2)
C3A—C4A—H4AA	119.7	C3B—C4B—H4BA	119.7
C5A—C4A—H4AA	119.7	C5B—C4B—H4BA	119.7
C6A—C5A—C4A	118.31 (17)	C6B—C5B—C4B	118.23 (19)
C6A—C5A—H5AA	120.8	C6B—C5B—H5BA	120.9
C4A—C5A—H5AA	120.8	C4B—C5B—H5BA	120.9
C5A—C6A—C1A	121.74 (16)	C1B—C6B—C5B	121.91 (17)
C5A—C6A—S1A	119.60 (14)	C1B—C6B—S1B	118.50 (14)
C1A—C6A—S1A	118.65 (13)	C5B—C6B—S1B	119.57 (14)
C9A—C7A—C8A	107.66 (15)	C9B—C7B—C8B	107.90 (15)
C9A—C7A—S1A	127.02 (14)	C9B—C7B—S1B	128.47 (14)
C8A—C7A—S1A	124.58 (13)	C8B—C7B—S1B	123.63 (13)
O3A—C8A—N1A	123.49 (16)	O3B—C8B—N1B	123.41 (16)
O3A—C8A—C7A	131.50 (16)	O3B—C8B—C7B	132.46 (16)
N1A—C8A—C7A	105.00 (14)	N1B—C8B—C7B	104.14 (15)
N2A—C9A—C7A	107.31 (15)	N2B—C9B—C7B	107.66 (16)
N2A—C9A—C10A	121.56 (15)	N2B—C9B—C10B	120.20 (16)
C7A—C9A—C10A	131.14 (16)	C7B—C9B—C10B	132.14 (16)
C9A—C10A—C11A	113.81 (14)	C9B—C10B—C11B	114.21 (15)
C9A—C10A—H10A	108.8	C9B—C10B—H10C	108.7
C11A—C10A—H10A	108.8	C11B—C10B—H10C	108.7
C9A—C10A—H10B	108.8	C9B—C10B—H10D	108.7
C11A—C10A—H10B	108.8	C11B—C10B—H10D	108.7
H10A—C10A—H10B	107.7	H10C—C10B—H10D	107.6
C13A—C11A—C12A	111.09 (17)	C13B—C11B—C12B	111.52 (17)
C13A—C11A—C10A	111.16 (15)	C13B—C11B—C10B	109.50 (16)
C12A—C11A—C10A	109.05 (16)	C12B—C11B—C10B	111.26 (16)
C13A—C11A—H11A	108.5	C13B—C11B—H11B	108.1
C12A—C11A—H11A	108.5	C12B—C11B—H11B	108.1
C10A—C11A—H11A	108.5	C10B—C11B—H11B	108.1
C11A—C12A—H12A	109.5	C11B—C12B—H12D	109.5
C11A—C12A—H12B	109.5	C11B—C12B—H12E	109.5
H12A—C12A—H12B	109.5	H12D—C12B—H12E	109.5
C11A—C12A—H12C	109.5	C11B—C12B—H12F	109.5
H12A—C12A—H12C	109.5	H12D—C12B—H12F	109.5
H12B—C12A—H12C	109.5	H12E—C12B—H12F	109.5
C11A—C13A—H13A	109.5	C11B—C13B—H13D	109.5
C11A—C13A—H13B	109.5	C11B—C13B—H13E	109.5
H13A—C13A—H13B	109.5	H13D—C13B—H13E	109.5
C11A—C13A—H13C	109.5	C11B—C13B—H13F	109.5
H13A—C13A—H13C	109.5	H13D—C13B—H13F	109.5
H13B—C13A—H13C	109.5	H13E—C13B—H13F	109.5
			
C8A—N1A—N2A—C9A	2.14 (19)	C8B—N1B—N2B—C9B	1.50 (19)
C6A—C1A—C2A—C3A	0.8 (3)	C6B—C1B—C2B—C3B	0.1 (3)
C1A—C2A—C3A—C4A	0.3 (3)	C1B—C2B—C3B—C4B	0.8 (3)
C2A—C3A—C4A—C5A	−1.3 (3)	C2B—C3B—C4B—C5B	−0.9 (3)
C3A—C4A—C5A—C6A	1.3 (3)	C3B—C4B—C5B—C6B	0.1 (3)
C4A—C5A—C6A—C1A	−0.2 (3)	C2B—C1B—C6B—C5B	−0.9 (3)
C4A—C5A—C6A—S1A	−178.87 (14)	C2B—C1B—C6B—S1B	177.62 (15)
C2A—C1A—C6A—C5A	−0.8 (3)	C4B—C5B—C6B—C1B	0.8 (3)
C2A—C1A—C6A—S1A	177.83 (15)	C4B—C5B—C6B—S1B	−177.67 (14)
O2A—S1A—C6A—C5A	−150.20 (15)	O1B—S1B—C6B—C1B	−176.39 (14)
O1A—S1A—C6A—C5A	−20.19 (17)	O2B—S1B—C6B—C1B	−47.59 (16)
C7A—S1A—C6A—C5A	94.35 (16)	C7B—S1B—C6B—C1B	66.43 (16)
O2A—S1A—C6A—C1A	31.11 (16)	O1B—S1B—C6B—C5B	2.15 (17)
O1A—S1A—C6A—C1A	161.12 (14)	O2B—S1B—C6B—C5B	130.95 (15)
C7A—S1A—C6A—C1A	−84.34 (15)	C7B—S1B—C6B—C5B	−115.03 (15)
O2A—S1A—C7A—C9A	−21.55 (18)	O1B—S1B—C7B—C9B	−20.70 (18)
O1A—S1A—C7A—C9A	−151.82 (15)	O2B—S1B—C7B—C9B	−150.22 (15)
C6A—S1A—C7A—C9A	93.73 (16)	C6B—S1B—C7B—C9B	95.44 (16)
O2A—S1A—C7A—C8A	169.54 (14)	O1B—S1B—C7B—C8B	159.51 (14)
O1A—S1A—C7A—C8A	39.28 (16)	O2B—S1B—C7B—C8B	29.99 (16)
C6A—S1A—C7A—C8A	−75.18 (16)	C6B—S1B—C7B—C8B	−84.35 (15)
N2A—N1A—C8A—O3A	175.65 (15)	N2B—N1B—C8B—O3B	177.71 (15)
N2A—N1A—C8A—C7A	−3.48 (18)	N2B—N1B—C8B—C7B	−1.81 (18)
C9A—C7A—C8A—O3A	−175.45 (17)	C9B—C7B—C8B—O3B	−177.96 (17)
S1A—C7A—C8A—O3A	−4.7 (3)	S1B—C7B—C8B—O3B	1.9 (3)
C9A—C7A—C8A—N1A	3.59 (18)	C9B—C7B—C8B—N1B	1.50 (18)
S1A—C7A—C8A—N1A	174.31 (12)	S1B—C7B—C8B—N1B	−178.68 (12)
N1A—N2A—C9A—C7A	0.25 (19)	N1B—N2B—C9B—C7B	−0.46 (19)
N1A—N2A—C9A—C10A	−179.75 (15)	N1B—N2B—C9B—C10B	179.60 (14)
C8A—C7A—C9A—N2A	−2.40 (19)	C8B—C7B—C9B—N2B	−0.66 (19)
S1A—C7A—C9A—N2A	−172.83 (13)	S1B—C7B—C9B—N2B	179.53 (13)
C8A—C7A—C9A—C10A	177.60 (16)	C8B—C7B—C9B—C10B	179.26 (17)
S1A—C7A—C9A—C10A	7.2 (3)	S1B—C7B—C9B—C10B	−0.5 (3)
N2A—C9A—C10A—C11A	−99.45 (19)	N2B—C9B—C10B—C11B	67.8 (2)
C7A—C9A—C10A—C11A	80.6 (2)	C7B—C9B—C10B—C11B	−112.2 (2)
C9A—C10A—C11A—C13A	71.2 (2)	C9B—C10B—C11B—C13B	−174.69 (16)
C9A—C10A—C11A—C12A	−166.01 (17)	C9B—C10B—C11B—C12B	61.6 (2)

### Hydrogen-bond geometry (Å, °)

**Table:**

*D*—H···*A*	*D*—H	H···*A*	*D*···*A*	*D*—H···*A*
N1A—H1NA···O3A^i^	0.79 (3)	2.05 (3)	2.816 (2)	164 (3)
N2A—H2NA···O3B^ii^	0.85 (4)	1.85 (4)	2.640 (3)	155 (2)
N1B—H1NB···O1A^iii^	0.86 (3)	2.10 (4)	2.733 (3)	130 (3)
N2B—H2NB···O3A^iii^	0.88 (4)	1.83 (4)	2.700 (3)	170 (2)
C5A—H5AA···O1B^iv^	0.93	2.47	3.256 (3)	143
C5B—H5BA···O2A	0.93	2.48	3.307 (3)	149
C10A—H10B···O3B^ii^	0.97	2.57	3.324 (3)	135
C10B—H10D···O1B	0.97	2.41	3.152 (3)	133

Symmetry codes: (i) −*x*+2, −*y*+2, −*z*+1; (ii) *x*+1, *y*+1, *z*; (iii) *x*, *y*−1, *z*; (iv) −*x*+1, −*y*+1, −*z*+1.
